# Supplementary material for: A fault-intrusion-tolerant system and deadline-aware algorithm for scheduling scientific workflow in the cloud
Source: PeerJ Comput Sci. 2021 Nov 2;7:e747. doi: 10.7717/peerj-cs.747 (PMC8576554; doi:10.7717/peerj-cs.747)
Supplement: Supplemental Information 6 [file peerj-cs-07-747-s006.zip › Myproject1.1/docs/org/cloudbus/cloudsim/power/models/package-use.html]

Uses of Package org.cloudbus.cloudsim.power.models


JavaScript is disabled on your browser.


- Overview
- Package
- Class
- Use
- Tree
- Deprecated
- Index
- Help

- Prev
- Next

- Frames
- No Frames

- All Classes

# Uses of Package org.cloudbus.cloudsim.power.models

- Packages that use org.cloudbus.cloudsim.power.models

  | Package | Description |
  | --- | --- |
  |  |  |
  | --- | --- |
  | org.cloudbus.cloudsim.examples.power |  |
  | org.cloudbus.cloudsim.power |  |
  | org.cloudbus.cloudsim.power.models |  |
- Classes in org.cloudbus.cloudsim.power.models used by org.cloudbus.cloudsim.examples.power

  Class and Description

  PowerModel

  The PowerModel interface needs to be implemented in order to provide a model of power consumption
  depending on utilization for system components.
- Classes in org.cloudbus.cloudsim.power.models used by org.cloudbus.cloudsim.power

  Class and Description

  PowerModel

  The PowerModel interface needs to be implemented in order to provide a model of power consumption
  depending on utilization for system components.
- Classes in org.cloudbus.cloudsim.power.models used by org.cloudbus.cloudsim.power.models

  Class and Description

  PowerModel

  The PowerModel interface needs to be implemented in order to provide a model of power consumption
  depending on utilization for system components.

  PowerModelSpecPower

  The abstract class of power models created based on data from SPECpower benchmark:
  http://www.spec.org/power\_ssj2008/
  If you are using any algorithms, policies or workload included in the power package, please cite
  the following paper:
  Anton Beloglazov, and Rajkumar Buyya, "Optimal Online Deterministic Algorithms and Adaptive
  Heuristics for Energy and Performance Efficient Dynamic Consolidation of Virtual Machines in
  Cloud Data Centers", Concurrency and Computation: Practice and Experience (CCPE), Volume 24,
  Issue 13, Pages: 1397-1420, John Wiley & Sons, Ltd, New York, USA, 2012

- Overview
- Package
- Class
- Use
- Tree
- Deprecated
- Index
- Help

- Prev
- Next

- Frames
- No Frames

- All Classes
